# Role of Melatonin on Virus-Induced Neuropathogenesis—A Concomitant Therapeutic Strategy to Understand SARS-CoV-2 Infection

**DOI:** 10.3390/antiox10010047

**Published:** 2021-01-02

**Authors:** Prapimpun Wongchitrat, Mayuri Shukla, Ramaswamy Sharma, Piyarat Govitrapong, Russel J. Reiter

**Affiliations:** 1 Center for Research and Innovation, Faculty of Medical Technology, Mahidol University, Nakhon Pathom 73170, Thailand; prapimpun.won@mahidol.ac.th; 2 Chulabhorn Graduate Institute, Chulabhorn Royal Academy, Bangkok 10210, Thailand; mshuklag@gmail.com; 3 Department of Cell Systems & Anatomy, University of Texas Health Science Center at San Antonio, San Antonio, TX 78229, USA; sharmar3@uthscsa.edu

**Keywords:** melatonin, Covid-19, neurodegeneration, viral infection, oxidative stress, reactive oxygen species, anti-inflammation

## Abstract

Viral infections may cause neurological disorders by directly inducing oxidative stress and interrupting immune system function, both of which contribute to neuronal death. Several reports have described the neurological manifestations in Covid-19 patients where, in severe cases of the infection, brain inflammation and encephalitis are common. Recently, extensive research-based studies have revealed and acknowledged the clinical and preventive roles of melatonin in some viral diseases. Melatonin has been shown to have antiviral properties against several viral infections which are accompanied by neurological symptoms. The beneficial properties of melatonin relate to its properties as a potent antioxidant, anti-inflammatory, and immunoregulatory molecule and its neuroprotective effects. In this review, what is known about the therapeutic role of melatonin in virus-induced neuropathogenesis is summarized and discussed.

## 1. Introduction

Neurodegenerative diseases are incurable and affect millions of people worldwide, leaving the individuals with debilitating health conditions. Alzheimer’s disease (AD) and Parkinson’s disease (PD) are the most common neurodegenerative diseases. Neurodegenerative diseases are progressive in nature and lead to degeneration and nerve cell death, ultimately causing a decline in both the central nervous (CNS) and peripheral nervous system (PNS) functions. Accumulation of intracellular misfolded and fibrillated proteins are major hallmarks of such diseases and involve the specific actions of endoplasmic reticulum (ER) stress, ubiquitin proteasome system (UPS), and phagosomes. Mutations in the mitochondrial DNA induce reactive oxygen species (ROS) production, leading to oxidative stress. Upregulation of the apoptotic signaling via the caspase cascade drives the progression of cell death [1].

Melatonin is a neurohormone produced in and secreted by the pineal gland, especially at night. It reaches the cerebrospinal fluid (CSF) either indirectly after its release into the blood and it subsequent discharge into the third ventricle, or, more likely, by being secreted directly into the ventricle by the pinealocytes [2], from where it reaches the cerebral tissues under physiological conditions, as evidenced by in vivo scintigraphy and autoradiography [3]; from the CSF, melatonin can more readily diffuse into the neural tissue where it protect neurons and glia from damage [4]. Melatonin is also likely produced in neurons and glial cells, which may contribute to the high levels of melatonin in the CSF compared to blood concentrations. Considering these findings, CSF-derived melatonin may decelerate the progression of neurodegenerative diseases [4]. Melatonin has a wide range of neuroprotective actions by modulating the pathophysiological mechanisms and signaling pathways observed in neurodegenerative diseases [5,6]. Along with its antioxidant, immunomodulatory, anti-inflammatory, and anti-apoptotic properties, melatonin also regulates abnormal protein dynamics, mitochondrial dysfunction and altered bioenergetics [7].

It is well documented that viral infections can lead to neurodegenerative changes, causing significant brain damage, along with neurobehavioral alterations. Viruses have evolved through their ability, over time, to impact the CNS by crossing the blood–brain barrier (BBB), leading to neuronal deficits and degeneration. The relationship between viral infections and brain disease dates back to 1385, when the link between psychosis and influenza infection was established, which became more apparent during the Spanish flu epidemic in 1918 [8] and perhaps more evident when a condition encephalitis lethargica was recognized viral antigens in the brains of infected people [9]. Additional evidence was provided when rats exposed to Japanese encephalitis virus (JEV) developed symptoms of PD [10]. Moreover, studies in infectious CNS disorders further revealed that viruses such as herpes and measles probably breach oligodendrocytes and compromise the immune system activity by altering normal cellular physiology, resulting in the demyelination of neurons, as observed in multiple sclerosis, in sclerosing panencephalitis and in neuromyelitis optica, which are chronic autoimmune demyelinating diseases [11]. Additionally, potential links have been established between herpes simplex virus (associated with *APOE-e4* allele) [12,13] and cytomegalovirus infection with inflammatory responses [14]. As human immunodeficiency virus (HIV) infection compromises the immune system, affected individuals become more susceptible to infections and diseases such as in HIV encephalopathy, a viral infection that causes neurodegenerative changes which are attributed to its ability to exploit the BBB, leading to dementia, which corresponds to the rate of spread of infection along with remarkable amyloid plaque formation [15]; this increases the risk of developing AD. Regarding PD, as the basal ganglia is an assailable target of HIV, infected subjects have a greater risk of developing this devastating condition [16]. In this context, apart from neurodegenerative diseases, much information is currently being gathered on investigations into the preventive roles of melatonin in patients affected by viral diseases like Covid-19 [17,18,19,20,21,22]. In this review, the therapeutic role of melatonin in virus-induced neuropathogenesis is discussed in detail. Additionally, how melatonin regulates and modulates the post-infection neurological complications is also evaluated. Therefore, based on the previous and current observations, we also emphasize that melatonin could prove to be a promising therapeutic approach in coping with Covid-19-induced neuropathological manifestations.

## 2. Neurological Syndromes Induced by Viral Infections

Clinically, neuronal viral infections are characterized as being acute or chronic. An acute viral infection is represented by the production of virions where the host immune system aids in eliminating the infection. Encephalitis, aseptic meningitis, encephalomyelitis, and flaccid paralysis are examples of acute neuronal viral infections, whereas viruses causing highly persistent infections add to the chronic neuronal viral infections, like retrovirus disease, spongiform encephalopathies, subacute-sclerosing panencephalitis and progressive multifocal leukoencephalopathy. The mechanism of entry of such viruses is either by breaching the BBB or entering the peripheral nerves through the axon, and thereafter being translocated to the cell body in the brain, where viral replication occurs.

### 2.1. Acute Viral Neurological Syndromes

#### 2.1.1. Encephalitis

Viruses such as arboviruses, herpes simplex and rabies are some of the viruses that cause pathological lesions in the grey matter characterized by focal neurological signs, convulsions, confusion, fever and altered consciousness [23].

#### 2.1.2. Aseptic Meningitis

A quite common viral infection confined to meninges is caused by enteroviruses and mumps virus and, less frequently, by herpes simplex virus type 2 (HSV-2) and varicella-zoster virus. Symptoms include fever and headache. Concerning the CSF findings, pleocytosis comprised of lymphocytes and polymorphs has been observed [24].

#### 2.1.3. Acute Flaccid Paralysis

Acute virus-induced neurological syndrome occurs when a virus spreads to the CNS via a hematogenous route and where the motor neurons in the spinal cord are directly infected and the patients develop fever and flaccid paralysis of muscles [25]. Polioviruses are the common causative viral agents.

#### 2.1.4. Encephalomyelitis-Post Infectious

White matter diseases, referred to as the post-infectious encephalitis, are characterized by demyelinating lesions in the brain and spinal cord. With an ambiguous etiology, they are considered to be T-cell-mediated auto-immune reactions. In comparison, Guillain–Barré syndrome (GBS) is a rare neurological disorder and is an immunological condition leading to demyelination of peripheral nerves [26]. From mild weakness, it can progress to complications that lead to severe paralysis associated with paresthesia.

### 2.2. Chronic Viral Neurological Syndromes

#### 2.2.1. Retrovirus Disease

Humans possess retroviruses that exist in two forms: as normal genetic elements in their chromosomal DNA (endogenous retroviruses) and as horizontally transmitted infectious RNA-containing viruses which are transmitted from human t -human (exogenous retroviruses, e.g., HIV and human T cell leukemia virus (HTLV)). HIV-associated neurocognitive disorder involves pathogenic mechanisms such as decreased proteostasis, DNA damage, vascular dysfunction and chronic inflammation, thus bolstering amyloid β (Aβ), tau, or α-synuclein accumulation, thereby leading to robust neurodegenerative changes [27,28]. HTLV is characterized by demyelinating lesions in both the brain and the spinal cord, where the host activates an immune response against the virus entering from either the peripheral blood or mucosal compartments [29].

#### 2.2.2. Subacute Sclerosing Panencephalitis (SSPE)

Sometimes referred to as Dawson disease, SSPE is caused by hypermutated measles virus, leading to progressive and chronic inflammation of the brain featuring the demyelination of nerve fibers [30].

#### 2.2.3. Progressive Multifocal Leukoencephalopathy

Progressive multifocal leukoencephalopathy is a rare fatal viral disease caused by the John Cunningham (JC) virus, characterized by progressive inflammation of the white matter at multiple sites, particularly that of the parietal and occipital lobes. A weakened immune system makes the host prone to this infection, leading to neurological disabilities including a decline in mental function and difficulty seeing and speaking. Demyelination of the nerve fibers impairs neurotransmission, where the degree of immunocompromise aligns with the myelin deterioration [31]. Treatment is aimed at reversing the immune deficiency to slow or stop the disease progression.

#### 2.2.4. Spongiform Encephalopathies

Slow virus infections are also known as prion diseases, after the presumed infectious agent, as well as transmissible spongiform encephalopathies (TSEs), after the histopathologic changes associated with these infections, and are members of a family of rare progressive neurodegenerative disorders [32]. They are distinguished by long incubation periods, characteristic spongiform changes associated with neuronal loss, and a failure to induce inflammatory response. The abnormal folding of the prion proteins leads to brain damage and the characteristic signs and symptoms of the disease. Additionally, sleep disruption is a prevalent clinical feature in human prion diseases, as evidenced in the case of the “eponymous” fatal familial insomnia, or an early-stage symptom as in certain types of Creutzfeldt–Jakob disease [33].

#### 2.2.5. Acute Disseminated Encephalomyelitis (ADEM)

ADEM is an uncommon inflammatory demyelinating disease of the CNS. ADEM is thought to be an autoimmune disorder characterized by a widespread attack of inflammation in the brain and spinal cord that damages myelin. White matter involvement predominates but gray matter can also be affected, particularly the basal ganglia, thalamus, and brainstem. Lesions may also be restricted to the brainstem or cerebellum. Tumor-like lesions also occur, and CSF presents pleocytosis [34]. ADEM often follows viral or bacterial infections, or less often, vaccination for measles, mumps, or rubella. For this reason, ADEM is sometimes referred to as post-infectious or post-immunization acute disseminated encephalomyelitis.

## 3. Viral Neuropathogenesis

### 3.1. BBB Invasion and Neuroimmune Interactions

BBB provides a natural protection against entry of all kinds of microbes into the brain. Viruses interact with the nervous system by breaking down the tight junctions by deregulation of the matrix metalloproteinase (MMP) activity and disrupting the actin cytoskeleton [35]. In some cases, a virus infects the endothelial cells and enters the brain via this route, where they activate an immune response and stimulate the induction of cytokines and chemokines, thus damaging the BBB. A Japanese encephalitis virus infection increases MMP-9 expression in a nuclear factor kappa-light-chain-enhancer of activated B cells (NF-κB), mitogen-activated protein kinase (MAPK)- and reactive oxygen species (ROS)-dependent manner in rat brain [36]. The complex interaction of viruses at the neuronal junctions involves and affects cytokine production, inflammation, oxidative stress signaling, expression of enzymes and proteasomal degradation. Interestingly, one of the important means of neuronal communication is by vesicles that carry messenger RNAs and allow many types of viruses to pass their genetic material from cell to cell [37,38], aiding in spreading toxic proteins, for example, Aβ, throughout the brain.

### 3.2. Virus Infection Induces Alzheimer’s Like Features

AD is a neurodegenerative disorder characterized by the progressive impairment of cognitive functions which are associated with synaptic and neuronal loss. Several studies have demonstrated that viral infections may induce hallmarks of AD in association with neuronal damage and the impairment of cognitive functions. Most of these studies reveal that latent infections from viruses such as HSV and HIV pose a major risk factor for AD by inducing alterations to the amyloid precursor protein (APP)-processing homeostasis [15,39]. HSV infection is life-long process where the virus typically resides latently in the trigeminal ganglia of the fifth cranial nerve. High levels of HSV have been reported in the brain of AD patients [40]. Reactivation of herpes simplex virus type 1 (HSV-1) enhances its ability to penetrate the BBB into the limbic system and areas of the brain most often affected in AD [41]. The alteration of APP processing induced by HSV-1 infection has potential causality in the development of AD. HSV-1 infection induces the amyloidogenic pathway by increasing the β-site APP-cleaving enzyme 1 (BACE1) and the γ-secretase subunit (nicastrin) [42]. Inhibition of non-amyloidogenic pathway by decreasing the activity of α-secretase is detected in HSV-1-infected neuronal cells [43]. HSV-1 provokes multiple cleavages of APP and promotes intracellular accumulation of various neurotoxic species including Aβ_40_ and Aβ_42_ [43,44,45].

HSV-1 also is associated with tau processing and the formation of phosphorylated tau protein and neurofibrillary tangles (NFTs), both in vitro and in vivo. Tau processing has been previously demonstrated to increase the kinetics of tau aggregation that can contribute to induction of neurodegenerative processes [46]. Levels of p-tau and its cleaved fragments are increased in mouse hippocampi and cortex after HSV-1 infection [47,48]. The mechanism involved in HSV-1-induced tau processing shows that HSV-1 triggers caspase-3 activity and induces cleavage of tau in neurons, which is considered a marker of early neurodegeneration [46,47]. For other viruses of the same family, *Herpesviridae*, in vitro studies show that HSV-2 infection leads to a prominent accumulation of Aβ_40_ and Aβ_42_ and induces tau phosphorylation in human SK-N-MC neuroblastoma cells. The reduction in secreted Aβ_40_, secreted APPα and the α-C-terminal fragment (α-CTF) after HSV-2 infection indicates the disruption of the APP nonamyloidogenic pathway and impairment of Aβ secretion in these cells [49]. A neurotropic HSV infection is a risk factor for the development of AD neurodegenerative hallmarks in the CNS in long-term infections.

HIV, a virus in the family *Retroviridae*, causes immunodeficiency which leads to acquired immune deficiency syndrome (AIDS). HIV infects the CNS and causes enhanced neurotoxicity that can directly damage the brain and is manifested as a set of abnormalities known as HIV-associated neurocognitive disorders (HAND) [50,51,52]. The deposition of Aβ is a common pathologic feature found in the brain of HIV positive patients with prolonged antiretroviral treatment and aging [53,54]. Moreover, the patterns of change in Aβ_42_, total tau, and p-tau levels in CSF of HAND cases are similar to those in AD [55]. The hyperphosphorylated tau is found in the hippocampus of HIV-1 patients and marked levels of hyperphosphorylated tau are noted in anti-retroviral therapy-treated subjects [55]. In HIV transgenic animals, HIV-1 tat protein induces tau phosphorylation via multiple mechanisms that lead to the formation of NFTs [56,57]. These results suggest that the neurotoxic viral protein could be a risk factor for subsequent AD- and/or HIV-related cognitive impairment.

### 3.3. Virus Infections That Induce Cognitive Impairment

Some of the neurotropic virus infections cause the impairment of cognitive function by altering neurotransmission. The cholinergic system has an important role in memory and learning. JEV infection not only induces neuronal damage [58,59,60], it also causes a reduction in acetylcholinesterase (AChE) activity and causes damage in the frontal cortex, midbrain, thalamus, hippocampus and cerebellum. JEV-infected rats exhibit a transient dysfunction of memory learning [61,62]. The reduction in total muscarinic cholinergic binding, cholinergic receptor muscarinic 2 (Chrm2) gene and choline acetyltransferase (ChAT) expression in different brain regions also correlates with transient spatial learning and memory impairment in JEV-infected rats [62].

Glutamate-mediated excitoneurotoxicity is an important mechanism of neuronal injury. JEV infection increases the concentration of glutamate and reduces the level of its NMDA receptors such as NR1, NR2A and NR2B in association with the redox imbalance in neural cells [63,64]. The increase in glutamate-mediated excitoneurotoxicity relates to the increase in oxidative damage in the brain, which is responsible for memory and learning impairment in JEV infection. In addition, the decreased levels of catecholamines including norepinephrine, dopamine, 3,4-dihydroxyphenylacetic acid, homovanillic acid, and serotonin are unrecoverable in the brain, which leads to the alteration of brain function including locomotor activity in JEV-infected rats [65]. This suggests that a neurotropic virus infection disrupts the level of neurotransmitters and causes an impairment in the regulation of specific brain functions relating to the neuropathogenesis in a virus infection.

Additionally, H5N1 influenza virus is capable of entering the brain through the BBB and easily infiltrates nerve cells to kill them; it especially targets the dopamine-producing neurons in the substantia nigra [66]. Conversely, the H1N1 flu strain is not capable of crossing the BBB, but still can cause the CNS immune cells to invade the substantia nigra and the hippocampus, causing inflammation and cell death in those areas [67,68]. The damage to the neurons in those areas reflects the cognitive dysfunction that occurs.

For the progression of the neural diseases, the most important factor is how the immune system of the host responds to the infection as humans have varied and complex antiviral defenses. Such interactions are capable of directly affecting the neuronal activities, thus diversifying the complexity of viral invasion and responses evoked by the CNS. For example, humans with C-C chemokine receptor type 5 (CCR5delta 32), a mutated chemokine receptor, show a reduced potential for developing AIDS and consequent AIDS-related dementia from HIV, whereas West Nile Virus (WNV) encephalitis is more common in people with the same mutated receptor [69]. In herpes, simplex encephalitis the virus invades the brain, affecting the temporal lobe, which controls memory and speech. Some reported mutations altering the susceptibility of herpes infections include those affecting Unc-93 homolog B1 (UNC)-93B, signal transducer and activator of transcription 1 (STAT1), and Toll-like receptor 3 (TLR3) [70,71]. After the initial activation of the innate neuronal and glial antiviral defenses in response to an infection, an upregulation of the virus-specific B, T, natural killer cells, along with macrophages, indicates the activation of acquired immunity. Many viruses have evolved an ability to block interferon-mediated cellular antiviral defenses. For instance, the Sindbis virus (SINV) and Venezuelan equine encephalitis virus (VEEV) block interferon responses by attenuating the phosphorylation of downstream STAT1 and STAT2 pathways [72]. STAT proteins are intracellular transcription factors that mediate many aspects of cellular immunity, proliferation, apoptosis, and differentiation. Once inside the nucleus, these viruses bind to a consensus DNA-recognition motif in the promoter region of the cytokine inducible genes, thus activating transcription.

### 3.4. Coronaviruses: Neuropathogenesis

Coronaviruses are a large family of RNA viruses that cause respiratory dysfunctions. Hundreds of such viruses have been identified thus far, of which Middle East Respiratory Syndrome (MERS) and Severe Acute Respiratory Syndrome (SARS) viruses are considered to cause the greatest damage. SARS-CoV-2 is a coronavirus affecting humans with the ability to transmit in diverse virulent forms. It has recently been recognized as the Covid-19 disease, which has already been considered a global pandemic. Covid-19 is an inflammatory disease associated with the dysregulation of the immune inflammatory response and altered immunomodulatory functions. Even though Covid-19 primarily affects the respiratory system, an increasing number of reports have affirmed neurological symptoms including neurodegenerative changes and mental health disorders [73,74]. Mounting evidence indicates that Covid-19 has substantial impacts on the CNS. Apart from robust neurochemical alterations, recent observations also include stroke and spinal cord complications. Diagnosing Covid-19 at an early stage marks the prognosis of this disease, where symptoms like anosmia and ageusia contribute to early diagnosis. A wide array of neurological manifestations has been meticulously examined in Covid-19 patients; they are discussed later in this report.

To date, an understanding of the underlying mechanisms of Covid-19-induced neurological complications documents the activation of the immune system and neuroinflammation, as shown by functional proteomic studies where elevated neuroinflammatory markers were present in the plasma and CSF of such patients [75]. As such, the cerebrovascular permeability and neuroinflammatory responses have been key mechanisms in the neurodegenerative changes post viral infections.

The nose and mouth are the major entry points for this virus, where the virus enters through the nose, reaching the olfactory bulb. A critical target at this juncture is the human angiotensin-converting enzyme-2 (hACE2) receptor with which the virus interacts and allows entrance. The presence of this receptor in the nasal mucosa, lungs and the brain contributes to its neuroinvasive nature; at this point, however, there is a question as to whether the olfactory nerve endings in the nose actually contain the ACE2 receptor.

Neuroimaging studies of a Covid-19 patient revealed a rare encephalopathy called associated acute necrotizing hemorrhagic encephalopathy (ANE), which leads to brain dysfunction with seizures and mental disorientation [76]. These findings indicate the presence of human ACE2 receptors in CNS. The brain may be infected by the virus not only through the olfactory bulbs, but also through other peripheral nerve terminals, or simply through blood circulation, to attack CNS nuclear groups in the medulla, specifically the nucleus solitarius, an area that receives taste information and coordinates gastrointestinal and respiratory neural networks and modifies the breathing pattern [77]; the latter may account for the acute respiratory failure in Covid-19 cases. Similar to Covid-19, earlier studies of SARS and MERS viral infections have reported that these viruses enter the brain through nerve cells that express ACE2, causing extensive brain damage and death. Thus, some patients undergo respiratory failure even with low levels of the virus in the lungs.

## 4. Roles of Melatonin on Controlling Viral Infections

Melatonin supplementation has been suggested to potentially have benefits against several viral infections from diverse families based on the results of both in vitro and in vivo experiments. Studies reporting the effects of melatonin on virus infections are summarized in Table 1. Melatonin exhibits its protective action in all tissues; for example, in the CNS, as elsewhere, it functions as a powerful free-radical scavenger with the ability to also regulate immune function. Furthermore, melatonin treatment has confirmed its actions against infections for both neurotropic and non-neurotropic viruses due to a variety of protective functions. In this context, melatonin was shown to exert:

(a) The antiviral effects are a result of reducing the viral titer or lowering new progeny production in the target tissues or cells by inhibiting the viral replication processes [78,79,80,81,82,83];
(b) The antioxidant effects limit free radical production and lipid peroxidation, subsequently reducing oxidative stress in virus-infected cells. Maintaining a balance in the redox reactions, together with the regulatory effects of the mitochondria and ER in virus-infected cells, promotes the survival of infected cells [82,83,84,85,86,87,88];
(c) The anti-apoptotic effects protect against virus-induced apoptotic cell death via the regulation of intrinsic apoptosis pathway, which increases the anti-apoptotic proteins while lowering proapoptotic proteins and caspase cascade activity [82,85,89,90];
(d) The regulatory effects on the autophagy pathway, which facilitates the clearance of cellular debris from virus-infected tissues [82,89];
(e) The immunomodulatory effects regulate and promote immune system responses to counteract the invasion of viruses [81,91,92,93];
(f) The anti-inflammatory effects inhibit the excessive inflammatory response resulting from virus infections to prevent the adverse effects of proinflammatory cytokines on the host cells [80,81,84,85,89,94,95];
(g) Postpone the onset and reduce the severity of the disease or death and promote a decline in mortality rates due to the virus infection [78,79,81,83,85,89,96,97,98,99].

### 4.1. Observations on the Role of Melatonin in Virus-Associated Encephalitis

Melatonin has beneficial actions especially on neurotropic viruses which reveal important cellular signaling mechanisms related to the protection of virus-induced neurodegeneration in the CNS. Melatonin reduces the viral load in the serum of mice infected with lethal Semliki Forest virus (SFV). SFV is an alphavirus belonging to the family *Togaviridae*. The virus is mainly spread through mosquito bites and causes pathology in the mouse CNS [100]. Administration of 500 µg/kg melatonin significantly delayed the onset of the disease and death in mice infected with SFV [78]. In addition, as documented in encephalomyocarditis virus (EMCV)-infected mice, the long-term treatment of melatonin was effective in reducing the severity of disease [99]. EMCV is the member of genus Cardiovirus in the family *Picornaviridae*. It is a non-enveloped, single-strand RNA virus that can infect a broad range of hosts via food or water contaminated with infected carcasses. The virus infection commonly damages the brain by inducing encephalitis and paralysis and can also cause myocarditis [101]. A daily injection of 1 µg of melatonin significantly reduced paralysis and decreased the mortality rate of EMCV-infected mice [99]. Another neurotropic virus, WNV, is a mosquito-transmitted virus in the family *Flaviviridae* that has neurovirulence by invading the CNS and inducing meningitis and encephalitis, which eventually cause neurological complications [102]. The induction of mortality by WNV infection in stress-exposed mice was reduced by melatonin administration [78]. These outcomes of the investigation documented the efficiency of melatonin in protecting against a lethal neurotropic virus infection.

The antiviral mechanisms of action of melatonin were further investigated in VEEV, a viral encephalitis infection. VEEV infection directly destroys the CNS, where it causes severe encephalitis, leading to permanent neurological sequelae [107,108]. Melatonin decreased the viral load in the brain and serum of mice infected with VEEV [79,83]. The administration of melatonin demonstrated its potential to suppress oxidative stress by decreasing nitric oxide (NO) production both in neuronal cells [86] and in the brain of infected mice [85,87]. Moreover, melatonin reduced the levels of malondialdehyde (MDA) and nitrite accumulation, indicating the reduction in the lipid peroxidation which is induced by VEEV infection [83,85]. The accumulation of VEEV in the brain causes inflammation and induces the production of pro-inflammatory factors in the infected brain [109,110]. Melatonin treatment diminishes the upregulation of CD200, a marker of inflammatory response expressed by VEEV, suggesting a potential anti-inflammatory effect of melatonin against VEEV infection [85]. Furthermore, long-term treatment with melatonin suggests the protective effect of melatonin on VEEV infection, involving a reduction in tumor necrosis factor-α (TNF-α) synthesis along with elevating the production of interleukin-1β (IL-1β) both in the blood and brain [91]. No differences, however, were detected in the levels of interleukin-2 (IL-2) and interleukin-4 (IL-4) [92]. IL-1β induced by melatonin treatment is a targeted cytokine which generates an immune response against the VEEV infection [92]. VEEV-infected mice treated with 500 μg/kg of melatonin displayed enhanced levels of serum interferon-γ (IFN-γ), which plays an important role in inducing and modulating an array of immune responses [92]. An increase in the host resistance to the virus via a peripheral immunostimulatory activity was considered responsible for these effects. Melatonin attenuates apoptotic cell death in infected cultured neuronal cells and in the brain of mouse models when induced by VEEV [85]. The reduction in cellular stress and inflammation after melatonin treatment documents the defense mechanisms and the survival of the cells during a VEEV infection. Giving supplementary melatonin to VEEV-infected mice led to reduced mortality rates, delayed onset of the disease and deferred time of death [83,85,96,98].

The findings summarized here confirm the neuroprotective effects of melatonin against neurotropic viruses and reveal a likely role for this neurohormone as an antiviral agent; it would seem to be a useful therapeutic agent for viral-induced neurodegenerative disorders. Effective drugs and treatments for viral disease could lower the mortality and reduce the impairment of the brain, which results in an increased quality of life for the patients.

### 4.2. Molecular Mechanism of Melatonin against Other Viruses

In addition to neurotropic encephalitis viruses, melatonin also suppresses nitroxative stress by lowering oxidants such as NO and·OH and increasing antioxidants including glutathione (GSH) synthesis and superoxide dismutase (SOD) activity in respiratory syncytial virus (RSV)-infected mice [84]. Melatonin reduces the production of pro-inflammatory cytokines in RSV infection by inhibiting inducible nitric oxide synthase (iNOS) expression and NF-κB/p65 activation, and downregulates the expression of TLR3 and TNF-α [84]. Upon co-treatment with IFN-γ, melatonin reduces a new viral progeny of human parainfluenza virus type 3 (HPIV3) in vitro [104]. Melatonin treatment decreases rabbit hemorrhagic disease virus (RHDV) titer in the liver of infected animals [80,82]. Reductions in glutathione disulfide (GSSG)/GSH ratio, endoplasmic reticulum (ER) stress and autophagy processes are observed in melatonin-treated RHDV-infected rabbits [82]. In addition, melatonin attenuates apoptotic liver damage induced by both intrinsic and extrinsic apoptotic pathways [82,95] and limited inflammation in fulminant hepatic failure in animal models which were induced by the RHDV; in this situation, melatonin also promoted regeneration in the liver [80,95].

A study conducted on coxsackievirus B3 (CVB3) infection, shows that melatonin administration prolongs the survival rate and reduces the severity of myocarditis and improves cardiac functions in the infected mice [89]. Moreover, melatonin exhibits anti-inflammation and anti-apoptosis effects by regulating the intrinsic apoptotic proteins through an increase in BCL-2 levels and reductions in BAX and caspase activity, together with the regulation of autophagic pathway; collectively, these changes promote the survival of the CVB3-infected cells [89]. Melatonin exhibits therapeutic potential in influenza A virus infection via its anti-inflammatory and immune modulatory effects [81]. Melatonin also has synergistic actions with ribavirin, an anti-viral drug, as shown by the increase in the survival of H1N1-virus-infected mice when compared to ribavirin alone [81]. The administration of melatonin to mink naturally infected with Aleutian mink disease virus (AMDV) increased the survival rate of infected animals [97]. Mice infected with the LP-BM5 leukaemia retrovirus develop murine AIDS; in these animals, melatonin stimulates the immune response, prevents the loss of vitamin E (an important immune modulator) and reduces excessive lipid peroxidation in splenocytes [93]. Additionally, melatonin has been screened for antiviral activity against dengue virus 2 (DENV2) [103].

Other effects of melatonin on viral infections have been reported to include the anti-tumor property on human papillomavirus (HPV) infection, as evidenced by the direct reduction in proliferation, migration, adhesion and viability of TC-1 tumor cell lines; this investigation used a combination of melatonin and an indoleamine 2,3-dioxygenase inhibitor [105]. Moreover, this combination also enhances vaccine-induced protective cellular immunity to HPV16-associated tumors [105]. In addition, melatonin was speculated to be an effective treatment for Ebola virus (EBOV) disease due to its protective ability in septic shock and its ability to decrease pro-inflammatory cytokines in several infectious pathogens [111]. In an elegant study in which a microvessel-on-a-chip system was treated with EBOV-like particles, which served as a model for Ebola hemorrhagic shock syndrome, it was demonstrated that melatonin effectively suppresses vasculopathy possibly via the modulation of the RHO/ROCK signaling pathway [106]. Thus, the studies on the role of melatonin in protecting against viral infections clearly demonstrate the potential antiviral property of this methoxyindole; it should be considered for development into a safe and effective anti-viral molecule.

### 4.3. Melatonin as a Potential Neuroimmune Modulator in Viral Infection

Besides being produced by the pineal gland, melatonin is also likely synthesized by the mitochondria of all cells [112,113,114,115], which itself could explain the pivotal role of melatonin in disorders pertaining to immune deficiencies. Melatonin’s intrinsic properties, including high cell permeability, the ability to easily cross the BBB and endogenous antioxidant properties, make it exceptionally beneficial for therapeutic purposes in neurological diseases. As with all blood vessels, brain microvascular endothelial cells are connected by tight junctions which restrict the entry of virus and bacterial particles into the CNS. Melatonin is well known for its role in preventing the permeability and maintaining the integrity of the BBB via its receptor activation as observed under inflammatory conditions in microvascular endothelial cells of rat brain [116]. By attenuating the loss of tight junctions and inhibition of MMP-9 [117], melatonin’s neuroprotective effects are preserved. It has also been demonstrated in methamphetamine-administered rats that melatonin prevents drug-induced structural impairments in the BBB, along with decrements in the pro-inflammatory cytokines such as IL-1β, interleukin-6 (IL-6), TNF-α, NF-κB and nuclear factor erythroid 2-related factor (Nrf2) signaling, which are involved in antioxidant defense [118]; these same actions have been documented in glioma cell lines [119] and human neuroblastoma cells [120].

One particularly important strategy used by viruses is to enter the PNS and travel in a retrograde manner in the axons to the CNS. The PNS consists of nerve fibers and ganglia that connect the CNS to peripheral tissues, which are protected from infection by peripheral innate and adaptive immune cells [121]. Another critical point of viral entry is through sensory and motor neurons that extend beyond the CNS barriers into the periphery. The differential expression of viral receptors on either sensory or motor neurons can dictate the type of peripheral nerve ending that a particular neurotropic virus will target. Poliovirus, adenoviruses and rabies virus bind to neurons at the neuromuscular junction owing to the neuronal expression of specific receptors. In this context, oxidative stress seems to be implicated in neuromuscular junction impairment, with mitochondrial dysfunction and inflammation being prominent features; related to this, melatonin has been reported to reverse age-related neuromuscular transmission dysfunction and improve muscle physiology [122,123].

Melatonin has an important regulatory role in the innate immunity which provides an early line of defense against pathogens [124]. By the cellular modulatory actions of melatonin on natural killer cells, macrophages, dendritic cells, neutrophils, etc., this indoleamine accounts for the early responses during the viral invasion in the nervous system. Melatonin modulates both pro- and anti-inflammatory cytokines in pathological conditions mediated by inflammation by regulating intracellular complexes, caspases and, in particular, the NLR family pyrin domain containing 3 (NLRP3) inflammasome [125], which recognizes pathogen-associated molecular patterns [126]. Melatonin has anti-viral actions [127] due to its anti-inflammatory and immune-enhancing characteristics on viral encephalitis infection [78,101,128,129] along with Ebola virus infection; for this purpose, a daily dose of about 40 mg melatonin has been suggested [130]. Immunomodulation is considered as a part of the treatment for ADEM [131] which further suggests the therapeutic potential of melatonin in this particular viral infection.

Both α- and β-adrenoceptors are expressed in the immune cells with this concurrent expression, providing a means to regulate inflammatory processes. In the microglia and in astrocytes, β2-adrenoceptor dysregulation contributes to neuroinflammation in autoimmune and neurodegenerative diseases [132]. In this context, melatonin regulates the expression of both α1- and β2-adrenoceptors in the hippocampus in rodents exposed to stressful conditions [133]. Viral infections produce a generalized immunodepression. Zhang Z et.al, 1999 [93], reported that treatment with dehydroepiandrosterone or melatonin, alone or in combination, prevented the reduction in B- and T-cell proliferation and the Th1 cytokine secretion caused by retroviral infection.

Melatonin acts as a buffer in immune interactions. During conditions of exaggerated immune responses, melatonin functions as an anti-inflammatory agent but under basal conditions or immunosuppressive status, it plays a stimulatory role [134]. Importantly, melatonin decreased the expression of iNOS protein [135], which is involved in immune responses when melatonin modulates calmodulin in a rhythmic manner to regulate many cellular functions. Moreover, melatonin also suppresses the activation of calcium/calmodulin-dependent kinase II (CaMKII) in axons and promotes nerve regeneration [136]. It is interesting to note that the host CaMKII inhibition is a potential target to develop prophylactic antiviral drugs against Dengue (DENV) and Zika virus (ZIKV) infections [137]. Melatonin exerts anti-inflammatory effects, mainly by inhibiting inflammasome activation and the caspase cascade [138]. In addition, it also contributes to its role as an antiapoptotic agent. Aging impacts both innate and adaptive arms of the immune system to impair control of viral infections, therefore the aged individuals are more prone to such infections [139]. Numerous studies support melatonin’s anti-aging role [140] in attenuating chronic and acute neuroinflammation, as evidenced in the brain of old rodents [141]. Via its immunomodulatory actions, melatonin acts on the immune system by regulating the cytokine production of immunocompetent cells, and downregulating adhesion molecules and pro-inflammatory cytokines, along with modification of serum inflammatory parameters [142]. Since melatonin is a powerful antioxidant, this neuroprotective molecule reduces oxidative stress remarkably, as observed in neurodegenerative disorders where brain oxidative damage has been implicated, thereby improving the clinical course of neural diseases.

Alterations in the innate and adaptive immune systems lead to an increased risk of infectious and degenerative diseases. A decrease in the CD4/CD8 ratio is also observed in elderly individuals among the major immune perturbations. Melatonin influences the activation and proliferation of CD4+ and naïve T cells in a time-dependent manner via promoting differentiation into helper T cells [143]. By regulating the activity of T-helper cells, melatonin counteracts the immunodeficiencies, thus protecting against lethal viral encephalitis and bacterial diseases [144]. Transcriptional factors of signal transducers and activators of transcription (STAT) proteins are redox-sensitive and participate in the regulation of cytokine signaling. Previous studies demonstrated that melatonin protects neurons through its antioxidative and anti-inflammatory effects in various neuropathological conditions. Melatonin exposure reduced oxidative stress and modulated STAT proteins, thus regulating cytokine signaling [145] in a rat model of traumatic brain injury (TBI). Melatonin administration reduced the concentration of free radicals and lipid peroxidation products in the serum and brain of encephalitis virus-infected mice [87], along with enhancing the efficiency of immunization against this virus [146]. Hence, its immunomodulatory actions, together with the antioxidant effects of melatonin, make this indoleamine an effective therapeutic agent to fight such viral diseases.

### 4.4. Melatonin Regulates Autonomic Nervous System in Viral Infection

Autonomic nervous system (ANS) has an important role in the regulation of homeostasis by modulating neuroinflammation, which affects overall the immune status in the neuronal circuitry where the vagus nerve transmits the impulse of peripheral inflammation to the brain stem. Both the sympathetic and the vagal efferent pathways participate in this anti-inflammatory and immunosuppressant action. The age-related changes which occur in the peripheral immune system also intensify the vulnerability to cognitive decline that is induced by infections. The all-inclusive nonspecific defense against pathogens is provided by the innate immune system, where epithelial cells forestall the entry of such pathogens, and the complementary system enhances the competence of the phagocytic cells and specific antibodies to counteract the microbes. The highly specific responses to the pathogen account for the adaptive immunity system involving the B and T lymphocytes [147]. An influenza virus infection in humans can lead to the development of a number of encephalitic syndromes with corresponding neurological consequences. The induction of inflammatory responses in the brain with the secretion of cytokines/chemokines and microglial activation has been observed in mice infected with A/Vietnam/1203/2004 (highly pathogenic avian H5N1 virus) and A/California/04/2009 H1N1 virus, suggesting a secondary CNS inflammation following the peripheral immune response [148]. It is notable that the autonomic responses are known to be mediated by the SCN and melatonin modulates the autonomic responses via activation of the central melatonin receptor signaling [149], as secretion of melatonin from the pineal gland is one of the effective mechanisms in the regulation of ANS [150]. Additionally, its age-associated decline also partially demonstrates how its levels are crucial in making the brain assailable to viral infections and correlated neurodegeneration.

## 5. Therapeutic Role of Melatonin on Post Infection Complications of Viral Induced Neuropathogenesis

### 5.1. Melatonin and Anosmia

Olfactory sensitivity exhibits a diurnal rhythm and the presence of melatonin receptor mRNAs and melatonin synthesis enzymes in the olfactory bulb affirms the possibility that melatonin is locally synthesized in the olfactory bulb [151]. Melatonin is widely known to inhibit neuronal apoptosis. In the olfactory bulb mucosae of rats, melatonin decreased the activity of caspase-3 and BAX and upregulated the expression of BCL-2, which is known to inhibit apoptosis by preserving the integrity of the mitochondrial membrane and which also inactivates BAX and other pro-apoptotic proteins [152]. Rodent studies have pointed out that GABAergic cells stimulate the production of interferons are essential for odor detection, where these cells exert a tonic inhibition that regulates the sensitivity of odor perception, as demonstrated in the mammalian brain [153]. Interestingly, modulation of the GABAergic system by melatonin might suggest the presence of a pathway for the neuroprotective effects of melatonin, given that melatonin impacts the circadian rhythm of endogenous GABAergic mechanisms [154,155]. Some anesthetic studies in humans have indicated a correlation between the levels of serum melatonin and olfactory identification impairment [156], which substantiates the involvement of melatonin in anosmia-like conditions.

### 5.2. Melatonin and Myelination

A study in a rodent model inoculated by the neuro-adapted John Howard Mueller (JHM) strain of mouse hepatitis virus (JHMV) has shown the development of an acute encephalomyelitis which disseminates throughout the brain parenchyma, causing demyelination and chronic neuroinflammation. The T cell chemoattractant chemokine CXCL10 (interferon-inducible protein IP-10) serves to recruit activated T and B lymphocytes expressing CXC chemokine receptor 3 (CXCR3), the receptor for CXCL10. Investigations have revealed that CXCL10 expression in the CNS is responsible for regulation of the JHMV viral replication and its uninterrupted expression leads to demyelination through CD4+ T-cell-induced enhancement of neuroinflammation via IFN-γ-mediated expression of other chemokines [157]. In this context, it has been reported that melatonin is involved in the regulation of CXCL10 production [158]. Melatonin also inhibits demyelination and increases remyelination, suggesting its local regulation in white matter astrocytes by serotonin availability and apolipoprotein E4 [159]. Melatonin stimulates oligodendroglia-enhanced remyelination by increasing pyruvate dehydrogenase kinases 4 (PDK4) expression and N-acetylaspartate (NAA) levels with a simultaneous reduction in inflammatory mediators in a mouse model of multiple sclerosis [160]. Moreover, multiple sclerosis is a chronic, progressive, inflammatory autoimmune disease of the myelin sheath. It has been recently documented that melatonin displays neuroprotective effects during both remyelination and demyelination stages in a rodent model of multiple sclerosis [161].

### 5.3. Melatonin and Myalgia

Myalgias are defined as muscle aches presenting as a sharp sporadic ache or a constant and deep ache. They are common in patients exposed to viral infections like coronaviruses and influenza. Nearly 30–40% of Covid-19 patients display myalgia as an early symptom [162,163]. The common underlying pathogenic mechanisms include oxidative stress and inflammation. In general, melatonin availability has been significantly associated with increased muscle strength in the average elderly population [164]. The results of one study confirmed the beneficial effects of melatonin in age-related muscle weakness [125]. Melatonin, received in a dose- and time-dependent manner, also reduced fibromyalgia-related musculoskeletal damage. Nonetheless, the therapeutic benefits of melatonin have not been thoroughly evaluated in several pain syndromes like fibromyalgia, headaches and chronic back pain [165].

### 5.4. Melatonin and Hypoxic Ischemia/Stroke

Hypoxic-ischemia often causes serious brain damage. Accumulating evidence has suggested that the neuroimaging features of hospitalized Covid-19 patients are usually dominated by acute ischemic infarction and intracranial hemorrhages [166]. Areas of the brain which may be preferentially affected by hypoxic-ischemic encephalopathy include the cerebral cortex, watershed regions between the anterior and middle cerebral artery and middle and posterior cerebral artery territories, basal ganglia, hippocampi, thalamus, cerebellum, and deep white matter [167]. Melatonin may potentially be useful in the treatment of neurodegenerative conditions that may involve free radical production, such as perinatal hypoxia. In this particular experiment, exogenously administered melatonin in animals effectively protected against brain injury by oxidative stress [168]. Increasing evidence has shown that, in animal stroke models, administering melatonin significantly reduces infarct volume, edema, and oxidative damage and improves electrophysiological and behavioral performance. Melatonin has shown neuroprotection in cerebral ischemia in acute, sub-acute, as well as chronic stages. In addition to its potent antioxidant properties, melatonin exerts antiapoptotic, antiexcitotoxic, anti-inflammatory effects and promotes mitochondrial functions in animals with cerebral ischemia [169]. Furthermore, in a neonatal rat model of stroke, melatonin increased myelin basic protein immunoreactivity in the cingulum along with increment in the mature oligodendrocytes, thus promoting myelination in the white matter with a subsequent reduction in inflammation [170]. Melatonin regulates the immunogenic cascade and inflammatory signaling in ischemic damage to the brain induced by stroke [171]. Clinical investigation in stroke patients revealed the therapeutic efficacy of melatonin administration by reduction in the oxidative response and increase in the survival rate of the patients in the intervention groups [172].

### 5.5. Melatonin and Prion Diseases

Viral infections can lead to prion diseases, which are pathological conditions associated with neuronal damage and associated behavioral disorders. Melatonin significantly alleviated PrP^c^ induced apoptosis and mitochondrial fragmentation and dysfunction, prevented the suppression of ATP and reduced the overproduction of ROS in N2A cell cultures, thus alleviating neuronal damage [173], which is suggestive of melatonin’s use in the treatment of prion diseases. Sleep disruption is a prevalent clinical feature in many neurodegenerative disorders, including human prion diseases. Fatal familial insomnia (FFI) is an inherited prion disease that mainly affects the thalamus. The thalamus is the part of the brain that controls the sleep–wake cycle, but is also known as the “relay center” of the brain because it helps the different parts of the brain communicate with each other [174]. A decrease in the levels of serum melatonin and corresponding disturbances in sleep patterns are observed in FFI patients [175]. Based on this information, it can be speculated that a planned timely daily dose of melatonin might improve sleep parameters in such patients.

### 5.6. Melatonin and Guillain–Barré Syndrome

Several reports have described Covid-19 patients as suffering from GBS [176]. GBS is triggered by a viral or bacterial infection where the body’s own immune system attacks the nerves. Symptoms start as weakness and tingling in the feet and legs that spreads to the upper body, and later, paralysis can also occur. GBS has several forms; of them, acute inflammatory demyelinating polyradiculoneuropathy (AIDP) is the most common, in which muscle weakness starts in the lower part of the body and spreads upward. Miller Fisher syndrome (MFS), in which paralysis starts in the eyes, and acute motor axonal neuropathy (AMAN) and acute motor-sensory axonal neuropathy (AMSAN), are less common forms. Sleep and psychiatric disturbances, along with anxiety, are common manifestations of GBS [177]. As such, low-level inflammation induced by neurotropic viruses increase the risk of psychiatric disorders [178]. Additionally, neuropathic pain also accounts for the neurological complication associated with this disorder [179]. It has been shown in preclinical studies that melatonin may be useful in the management of neuropathic pain [180]. Disturbances in the melatonin secretion is a characteristic feature in many psychiatric disorders [181] and exogenous melatonin has proven useful in patients with varied forms of psychiatric disturbances [182].

### 5.7. Melatonin and Neurotransmitters

Virus-induced pathogenesis also involves glutamate dysfunction, leading to brain damage [183]. Human coronavirus strain OC43 affects human glial and neuronal cells. Expression of the glial glutamate transporter 1 is responsible for glutamate homeostasis, which has been shown to decrease following this viral infection. One report demonstrated that inhibition of glutamate excitotoxicity using a 2-amino-3-(5-methyl-3-oxo-1,2-oxazol-4-yl)propranoic acid (AMPA) receptor antagonist (GYKI-52466) improved clinical scores related to paralysis and motor disabilities in S mutant virus-infected mice, as well as protecting the CNS from neuronal dysfunctions [178]. Melatonin has dynamic neuroprotective properties. In an HT22 mouse hippocampal cell line, melatonin reduced glutamate-induced oxytosis via its antioxidant action targeted at the mitochondria [184]. Recently, targeting glutamate with melatonin has been discussed for its possible therapeutic benefits in the treatment of anxiety [185], which is yet another complication observed in virus-infected patients.

Viruses such as wild type vesicular stomatitis virus (VSV) can rapidly and selectively infect and destroy serotonin neurons in young mice and rats. A subsequent immune system-mediated response eliminates all trace of the virus from the brain, leaving a permanent reduction in serotonin neurons, with consequent behavioral alterations [186], with no viral element remaining. The neurotransmitter and behavioral changes resulting from this initial infection may last for the lifetime of the organism, despite the lack of any trace of the virus in the brain, and with little detectable viral-mediated neuropathology evident by standard screening methods at the end of life. In this context, it has been shown that melatonin regulates (via its deacetylation to 5-methoxytryptamine) CYP2D-mediated synthesis of serotonin from 5-methoxytryptamine in the brain regions; hippocampus, cortex, striatum, nucleus accumbens, hypothalamus and thalamus as well as the medulla oblongata and cerebellum of the male Wistar rat, which are well recognized as the targeted pharmacological action of melatonin [187].

### 5.8. Melatonin and Autophagy

A variety of the stressful stimuli, which are triggered during different stages of viral replication, can induce autophagy. Virus-induced autophagy is capable of preventing the early apoptotic death of cells, suggesting that xenophagy, a cellular process dedicated to engulfing and destroying pathogens, might limit the cytopathic effect of viruses and the pathological consequences associated with cell death triggered by viral infection [188]. Viral infections trigger several processes, such as interaction with cell surface receptors and autophagic adaptors and oxidative and ER stress induction. These mechanisms can induce autophagy, which is inhibited at early or late stages of the pathway by certain viruses to promote its replication. The role of melatonin as a regulator of autophagy [189], due to its properties as a potent antioxidant and suppressor of ER stress [7], supports the potential beneficial role of this molecule in the management of some viral infections. Autophagy can be indirectly regulated by transcriptional mechanisms in response to the ROS/RNS via Nrf2/Keap1 pathway. Protection against the oxidant-induced exacerbation of influenza A virus infection is led by Nrf2-mediated antioxidant pathways [190]. The mechanisms by which melatonin stimulates the activities of ROS detoxifying enzymes involve the Nrf2/Keap1/ARE pathway [191], indicating that melatonin can regulate oxidative stress-induced autophagy via Nrf2. Additionally, melatonin also enhanced autophagy in senescence-induced neuronal cells via sirtuin 1 deacetylation of the RelA/p65 subunit of NF-κB [192].

### 5.9. Covid-19 and Melatonin Treatment

Based on the clinical studies conducted to date, it is now clear that Covid-19 disease induces severe neuropathological manifestations. Concerning the symptoms, headaches, dizziness and consciousness impairment accompanied by swelling of brain tissues and blood vessels with acute cerebrovascular disease symptoms account for the CNS-related symptoms, whereas PNS symptoms include anosmia, ageusia, vision impairment and neuropathic pain. It has been suggested that infection of the olfactory neurons in the nose enable the spread of the virus from the respiratory tract to the blood, after which they cross the BBB to enter the brain where the virus replicates and cause neurological disorders. The neurological complications in critically ill patients with Covid-19 were present in a case of meningitis/encephalitis associated with SARS-CoV-2 [193]. Only two types of human coronaviruses, namely HCoV-OC43 and E299, have been found to be neuroinvasive and can spread from the respiratory tract to the CNS [194]. The recent detection of SARS-CoV-2 in the CSF and in the frontal lobe sections from postmortem examination has confirmed the presence of the virus in neural tissue. Therefore, adequate levels of melatonin in the CSF have a crucial role to play in combatting the further spread of the virus. Most importantly, the presence of melatonin receptor mRNAs in the olfactory bulb itself shows that melatonin has an imperative role in modulating the virus action, from the entry level to the neuropathological manifestations post-infection.

Moreover, melatonin levels in the pineal gland and mitochondria correlate with the intensity of viral replication severity. Therefore, the scope of melatonin use in Covid-19 treatment has broadened [195]. Additionally, with the capacity of enhancing sirtuins which are considered as antiviral agents [196,197], melatonin may likely be an effective antiviral agent. The deadly outbreak of Covid-19 imposes immense therapeutic challenges. Interestingly, based on positive outcomes in many viral infections, melatonin therapy for viral infections like Covid-19 seems promising [198,199]. Clinically, it has been shown that melatonin has a therapeutic potential even in cases exhibiting obesity or diabetes in coexistence with Covid-19 [200]. An overreaction of the body’s immune system results in a massive production of inflammatory cytokines [201], and with anti-inflammatory, immunomodulatory properties, melatonin has proven effective in inhibiting the pathological manifestations in cases with viral infections in humans [130]. Melatonin not only counteracts the exaggerated cytokine response triggered by the virus but might also play a preventive role in regulating hypercoagulapathy, as seen in the blood vessels throughout the body and the brain in severe cases of Covid-19. This assumption is probably based on a study on melatonin and it was found that melatonin could diminish fibrinogen and elevate C-reactive protein (CRP) levels, regulate MDA and platelet morphology, and decrease prothrombin activity [202].

A network-based drug evaluation for Covid-19 has been formatted for therapeutic guidelines [203]. Selective estrogen receptor modulators have been considered because an overexpression of the estrogen receptor has been shown to play a crucial role in inhibiting viral replication [204]. Interestingly, the interaction between melatonin and estrogen, where melatonin has modulatory actions on this hormone [205], emphasizes what a pivotal role melatonin could play in Covid-19 therapeutics. Next, the angiotensin receptor blockers are suggested. In this context, melatonin administration has proven to be protective against angiotensin-II-induced vascular endothelial damage [206]. Furthermore, by binding to calmodulin and modulating the nAChRs, melatonin regulates the expression of ACE2, which could be beneficial in the context of Covid-19-associated neurotropism [207]. Immunosuppressant or antineoplastic agents are also considered as a part of a treatment strategy in combating viral infections. Melatonin’s immunomodulatory function [208] and its role as an antineoplastic [209] agent has been well acknowledged. Moreover, the anti-inflammatory actions [141] of melatonin and modulation of immune system of the host aids in the recovery and, eventually, eradication of the virus. The ubiquitin–proteasome system is involved in the early viral replicative cycle. Interestingly, like a proteasome inhibitor [210], melatonin may regulate several events involved in proteostasis [7].

A pharmaceutical composition for intranasal administration, consisting of melatonin (in the range of 50–1000 µg and in a single or simultaneous intranasal administration) and a pharmaceutically acceptable excipient, which causes the blood melatonin levels of human adult to rise, could be extremely beneficial to enhance immune response to Covid-19. Intranasal melatonin niosomes are bioequivalent to intravenous injection of melatonin, providing therapeutic level doses, and can deliver melatonin to the brain and peripheral tissues [211]. For humans infected with Covid-19, no definitive dosage scheme has been established, but the reader may want to consult the suggestions by authors who have published reports on this issue [212,213]. Melatonin can be administered via a variety of routes and nanoformulations have been suggested as potential improvements in melatonin’s efficacy, since they can be targeted to the mitochondria [214].

Contemporary research on Covid-19 has suggested that apart from being a complex disease, its systemic nature results in it affecting multiple cell types and organs, which calls for an effective treatment. A strategy like melatonin therapy could possibly counter the broad-spectrum disease manifestations [215]. Recently, a number of clinical trials are being conducted, aiming for melatonin to be included in the list of viable treatment strategies for Covid-19. Intravenous melatonin administration in ICU patients suffering from Covid-19 is currently under trial to better understand the dosage paradigm and efficaciousness of melatonin [18,22]. Another clinical trial is also under investigation, where the capability of melatonin will be explored in the prophylaxis of Covid-19 infection [20], along with its safety parameters compared to the standard therapeutic regimen [19]. Interestingly, melatonin treatment has positive outcomes in Covid-19 patients needing mechanical ventilation [21]. Supporting this notion, another study reviewed the potential beneficial effects of melatonin for the prevention and treatment of Covid-19 [216]. However, meticulous and extensive investigations would apparently promote the approval of the use of melatonin in the mainstream therapeutic regime for a complex infectious disease like Covid-19.

## 6. Conclusions

Virus infection affects the nervous system by interfering with the cellular mechanisms that could induce the neuronal degeneration. There are several published reports regarding the potential antiviral property of melatonin, which is suggestive of the utility of melatonin as a treatment for neuropathological manifestations induced by viral infections. It is now well accepted that Covid-19 disease induces severe neuropathological manifestations. Melatonin has a wide range of neuroprotective actions by modulating pathophysiological mechanisms via their antioxidant, immunomodulatory properties, and anti-inflammation, and anti-apoptotic properties. Based on positive outcomes in many viral infections, melatonin therapy for viral infections such as Covid-19 seems promising. The deadly outbreak of Covid-19 imposes immense therapeutic challenges which are being met with a number of different approaches. Collectively, the data regarding the use of melatonin to combat this infection seem to hold significant promise.

## Figures and Tables

**Table 1:** The Summary Effects of Melatonin against Virus Infection both in vitro and in vivo.

Family	Virus	Properties of Melatonin	Action of Melatonin	Dose of Melatonin	Dose of Virus	Model	Ref.
*Togaviridae*	Venezuelan equine encephalomyelitis virus (VEEV)	Antiviral effect	↓ Virus titer in supernatant	0.5, 1 and 5 mM	MOI 1 (Guajira strain)	Murine Na2 neuroblastoma cell line	[83]
			↓ Virus titer in brain	500 µg/kg (pre- and post-treatments)	10 PFU (Goajira strain)	NMRI mice	[83]
			↓ Virus titer in blood and brain	250, 500 and 1000 µg/kg (pre- and post-treatments)	100 PFU (Guajira strain)	NMRI-IVIC mice	[79]
		Antioxidant activity	↓ NO	0.025, 0.1, 0.25, 0.50, 1 and 1.8 mM	MOI 1 (Enzootic strain E-100)	Murine Na2 neuroblastoma cells	[86]
				100 and 150 µg/mL	(Guajira Strain)	Murine splenocyte	[88]
			↓ NO in blood and brain	500 µg/kg (pre- and post-treatments)	10 LD_50_ (Guajira strain)	NMRI mice	[85,87]
		Reduction of lipid peroxidation	↓ MDA	0.1, 0.5 and 1 mM	MOI 1 (Guajira strain)	Murine Na2 neuroblastoma cell line	[83,85]
			↓ MDA in brain	500 µg/kg (pre- and post-treatments)	10 PFU (Guajira strain)	NMRI mice	[83,85]
			↓ Nitrite ↓ iNOS	0.025, 0.1, 0.25, 0.50, 1 and 1.8 mM	MOI 1 (Enzootic strain E-100)	Murine Na2 neuroblastoma cells	[86]
		Anti-apoptotic effects	↓ Apoptotic cell death	0.1, 0.5 and 1 mM	MOI 1 (Guajira strain)	Murine Na2 neuroblastoma cell line	[85]
			↓ Apoptotic cell death in brain	500 µg/kg (pre- and post-treatments)	10 PFU (Guajira strain)	NMRI mice	[85]
		Immunomodulatory effects	↑ IL-1β in blood and brain, ↑ TNF-α and IFN-γ in blood	500 µg/kg (pre- and post-treatments)	10 LD_50_ (Guajira strain)	NMRI-IVIC mice	[87,91,92]
		Anti-inflammatory effects	↓ TNF-α in brain	500 µg/kg (pre- and post-treatments	10 LD_50_ (Guajira strain)	NMRI-IVIC mice	[91]
			↓ CD200 in brain	500 µg/kg (pre- and post-treatments)	10 PFU (Guajira strain)	NMRI mice	[85]
		Prolongs survival rate	↑ Survival rate of infected animals	Light-induced melatonin production (400 and 2500 lux)	100 PFU (Guajira strain)	NMRI-IVIC mice	[98]
			↑ Survival rate of infected animals ↓ Onset of the disease	250, 500 and 1000 µg/kg (pre- and post-treatments)	100 PFU (Guajira strain)	NMRI-IVIC mice	[79]
			↑ Survival rate of infected animals	500 µg/kg (pre- and post-treatments)	10 PFU (Goajira strain)	NMRI mice	[83,85]
			↑ Survival rate of immunodepressed infected animals	500 µg/kg (pre- and post-treatments)	100 PFU (Guajira strain)	NMRI-IVIC mice	[96]
	Semliki Forest virus (SFV)	Antiviral effect	↓ Virus titer in blood	500 µg/kg (pre- and post-treatments)	10, 100 PFU	CD1 mice	[78]
		Prolongs survival rate	↓ Onset of disease ↑ Survival rate of infected animals	500 µg/kg (pre- and post-treatments)	10, 100 PFU	CD1 mice	[78]
*Picornaviridae*	Encephalomyocarditis virus (EMCV)	Prolongs survival rate	↑ Survival rate of infected animals ↓ Paralysis	1 µg/day	2 × 10^8^ dilutions of virus	BALB/c mice	[99]
	Coxsackievirus B3 (CVB3)	Anti-apoptotic effects	↓ BAX, cleaved caspase-9 and cleaved caspase-3 in heart ↑ BCL-2 and BCL-2/BAX ratio in heart ↓ Apoptotic cells in heart	14.4 mg/kg/day	10^5.5^ TCID_50_	BALB/c mice	[89]
		Regulation of autophagic effects	on day 7 (heart) ↑ Autophagosomes ↑ LC3-II/LC3-I ratio and beclin-1 ↓ p62 on day 14 (heart) ↓ Autophagosomes ↓ LC3-II/LC3-I ratio and beclin-1 ↑ p62	14.4 mg/kg/day	10^5.5^ TCID_50_	BALB/c mice	[89]
		Anti-inflammatory effects	↓ TNF-α and IL-1 in heart ↓ Inflammatory cell infiltration, necrosis, and interstitial edema in myocardial tissue	14.4 mg/kg/day	10^5.5^ TCID_50_	BALB/c mice	[89]
		Prolongs survival rate	↑ Survival rate of infected animals ↓ Severity of the myocarditis ↑ Cardiac Function	14.4 mg/kg/day	10^5.5^ TCID_50_	BALB/c mice	[89]
*Flaviviridae*	West Nile virus (WNV)	Prolongs survival rate	↑ Survival rate of stress-exposed animals	5 µg/mouse (pre- and post-treatments)	2 × 10^5^ PFU (WN-25 attenuated variant)	CD1 mice	[78]
	Dengue virus type 2 (DENV2)	Antiviral effect	No effects	50 and 500 μM	MOI 2 and 5 (strain 16681)	Human hepatocellular carcinoma HepG2 cell line	[103]
*Orthomyxoviridae*	Influenza A virus (IAV)	Antiviral effect	↓ Virus titer in supernatant (co-treatment with ribavirin)	0.1, 0.25 and 0.5 mM	MOI 0.5 (H5N1)	Human lung adenocarcinoma A549 cell line	[81]
		Immunomodulatory effects	↑ IL-27, IL-10 and TGF-β in bronchoalveolar lavage fluid	20, 200 mg/kg (pre- and post-treatments)	1000 PFU (H5N1)	BALB/c mice	[81]
		Anti-inflammatory effects	↓ TNF-α, IL-6, IFN-γ and p-NF-κB in bronchoalveolar lavage fluid ↓ Th1 CD4 cells and Inflammatory CD8 T cells in spleen	20, 200 mg/kg (pre- and post-treatments)	1000 PFU (H5N1)	BALB/c mice	[81]
		Prolongs survival rate	↑ Survival rate of infected animals	20, 200 mg/kg (pre-and post-treatments)	1000 PFU (H5N1)	BALB/c mice	[81]
			↑ Survival rate of infected animals (co-treatment with ribavirin)	200 mg/kg/day	1000 PFU (H5N1)	BALB/c mice	[81]
*Paramyxoviridae*	Respiratory syncytial virus (RSV)	Antioxidant activity	↓ NO, MDA and ·OH in lung ↑ GSH and SOD in lung	5 mg/kg (orally administered twice daily for 3 days)	1 × 10^7^ PFU/mL (Long strain)	BALB/c mice	[84]
		Anti-inflammatory effects	↓ TLR3, NF-κB/p65 and TNF-α ↓ iNOS	10^−7^, 10^−6^ and 10^−5^ M	MOI 1 (Long strain)	Mouse macrophage RAW264.7 cell line	[94]
			↓ TNF-α in blood	5 mg/kg (orally administered twice daily for 3 days)	1 × 10^7^ PFU/mL (Long strain)	BALB/c mice	[84]
	Human parainfluenza virus type 3 (HPIV3)	Antiviral effect	↓ Virus titer in supernatant (co-treatment with IFN-γ)	0.05, 0.25 and 0.49 mM	MOI 1	Human lung adenocarcinoma A549 cell line	[104]
*Caliciviridae*	Rabbit hemorrhagic disease virus (RHDV)	Antiviral effect	↓ RHDV VP60 in liver	20 mg/kg at 0, 12, and 24 hpi.	2 × 10^4^ HA units	New Zealand white rabbits	[80,82]
		Antioxidant activity	↓ GSSG/GSH ratio in liver	20 mg/kg at 0, 12, and 24 hpi.	2 × 10^4^ HA units	New Zealand white rabbits	[82,90]
			Suppression of ER stress in liver ↓ CHOP ↓ BiP/GRP78	20 mg/kg at 0, 12, and 24 hpi.	2 × 10^4^ HA units	New Zealand white rabbits	[82]
		Anti-apoptotic effects	**Extrinsic apoptosis pathway (liver)** ↓ TNF-R1 ↓ p-JNK and caspase-8 ↑ c-FLIP **Intrinsic apoptosis pathway (liver)** ↓ BAX, cytosolic Cyt c and caspase-9 ↑ BCL-2 and BCL-xL ↓ caspase-3 and PARP-1	10 and 20 mg/kg at 0, 12, and 24 hpi.	2 × 10^4^ HA units	New Zealand white rabbits	[82,90]
		Regulation of autophagic effects	↓ LC3-II/LC3-I ratio, p62/SQSTM1, beclin-1, Atg5, Atg12 and Atg16L1 in liver	20 mg/kg at 0, 12, and 24 hpi.	2 × 10^4^ HA units	New Zealand white rabbits	[82]
		Anti-inflammatory effects	↓ TLR4, HMGB1, TNF-α, IL-1β, IL-6, CRP, MMP-9, SphK1/S1P, NF-κB p50 and p65 subunits and p-IκB-α in liver ↑ DAF/CD55 in liver	10 and 20 mg/kg at 0, 12, and 24 hpi.	2 × 10^4^ HA units	New Zealand white rabbits	[82,95]
		Stimulation of regenerative mechanisms	↑ HGF/c-Met, EGF/EGFR, PDGF-B/PDGFRβ, VEGF/VEGFR in liver ↓ p-JAK in liver ↑ ERK and STAT3 in liver	10 and 20 mg/kg at 0, 12, and 24 hpi.	2 × 10^4^ HA units	New Zealand white rabbits	[95]
*Parvoviridae*	Aleutian mink disease virus (AMDV)	Prolongs survival rate	↑ Survival rate of infected animals	2.7 mg melatonin crystals homogeneously suspended in medical grade silastic polymer	Natural infection	Mink	[97]
*Retroviridae*	Murine leukemia virus (MLV)	Immunomodulatory effects	Splenocyte ↑ B cell and T cell proliferation ↑ IL-2 and IFN-γ ↓ IL-4, IL-6 and IL-10 and TNF-α	49.8 µg/mouse/day	4.5 log_10_ PFU/mL (LP-BM5 retrovirus)	C57BL/6 mice	[93]
			↓ The loss of hepatic Vitamin E	49.8 µg/mouse/day	4.5 log_10_ PFU/mL (LP-BM5 retrovirus)	C57BL/6 mice	[93]
		Reduction of lipid peroxidation	↓ Hepatic conjugated dienes	49.8 µg/mouse/day	4.5 log_10_ PFU/mL (LP-BM5 retrovirus)	C57BL/6 mice	[93]
*Papillomaviridae*	Human papillomavirus (HPV)	Anti-tumor	↓ Proliferation, migration, adhesion and viability of tumor cells ↓ HPV-16 E6 and E7 oncoproteins	1 mM	HPV-16 genome encodes oncoproteins	TC-1 murine tumor cell line	[105]
		Improved vaccine efficacy	Co-treatment with DL-1MT ↑ Anti-tumor protective effects of gDE7 vaccine ↑ IFN-γ producing CD8+ T cells ↓ Tumor growth	0.2 mg/200 μL/mouse for 4 weeks every 48 h	Transplanted with TC-1 cells encoding HPV-16 oncoproteins	C57BL/6 mice	[105]
*Filoviridae*	Ebola virus (EBOV)	Protection of the integrity of blood vessels	↓ Rho/ROCK signaling ↓ Vascular permeability	0, 0.1, 1, 10 and 100 µM for 2 h	1 mg/mL of Ebola virus-like particles	Micro vessel (chip-based assay)	[106]

Abbreviations: Autophagy-related protein (ATG); Bcl-2-associated X protein (BAX); B-cell lymphoma 2 protein (BCL-2); Immunoglobulin heavy-chain-binding protein (BiP); Cellular FLICE-inhibitory protein (c-FLIP); CCAAT-enhancer-binding protein homologous protein (CHOP); Tyrosine–protein kinase Met (C-Met); Complement receptor 3 (CR3); C-reactive protein (CRP); Cytochrome *c* (Cyt *c*); Decay accelerating factor (DAF); 1-methyl-DL-tryptophan (DL-1MT); Epidermal growth factor (EGF); Epidermal growth factor receptor (EGFR); Extracellular mitogen-activated protein kinase (ERK); Glucose-regulated protein 78 (Grp78); Hepatocyte growth factor (HGF); High-mobility group box 1 (HMGB1); Heme oxygenase 1 (HO-1); Interferon γ (IFN-γ); Nitric oxide synthase (iNOS); Monocyte chemoattractant protein 1 (MCP-1); Histocompatibility complex (MHC); Anti-p21-activated kinase 1 (PAK-1); Poly(ADP-ribose)polymerase-1 (PARP-1); Platelet-derived growth factor (PDGF); Platelet-derived growth factor receptor (PDGFR); Phosphorylated inhibitor of kappa B-α (p-IκB-α); Phosphorylated Janus kinase (p-JAK); Rho-associated coiled-coil containing protein kinase (ROCK); Sphingosine-1-phosphate (S1P); Sphingosine kinase 1 (SphK1); Sequestosome 1 (SQSTM1); Tissue factors (TF); Tansforming growth factor β (TGF-β); T helper 1 (Th1); T helper 2 (Th2); Tumor necrosis factor receptor 1 (TNF-R1); Tissue factor pathway inhibitor (TPPI); Vascular endothelial growth factor (VEGF); Vascular endothelial growth factor receptor (VEGFR). ↑ Increase; ↓ Decrease.

## Data Availability

Not applicable.
